# Short-term effects of prosocial video games on aggression: an event-related potential study

**DOI:** 10.3389/fnbeh.2015.00193

**Published:** 2015-07-24

**Authors:** Yanling Liu, Zhaojun Teng, Haiying Lan, Xin Zhang, Dezhong Yao

**Affiliations:** ^1^The Lab of Mental Health and Social Adaptation, Faculty of Psychology, Research Center of Mental Health Education, Southwest UniversityChongqing, China; ^2^Key Laboratory for NeuroInformation of Ministry of Education, School of Life Science and Technology, Center for Information in Medicine, University of Electronic Science and Technology of ChinaChengdu, China

**Keywords:** prosocial video games, general learning model, event-related potential, P300, aggression

## Abstract

Previous research has shown that exposure to violent video games increases aggression, whereas exposure to prosocial video games can reduce aggressive behavior. However, little is known about the neural correlates of these behavioral effects. This work is the first to investigate the electrophysiological features of the relationship between playing a prosocial video game and inhibition of aggressive behavior. Forty-nine subjects played either a prosocial or a neutral video game for 20 min, then participated in an event-related potential (ERP) experiment based on an oddball paradigm and designed to test electrophysiological responses to prosocial and violent words. Finally, subjects completed a competitive reaction time task (CRTT) which based on Taylor's Aggression Paradigm and contains reaction time and noise intensity chosen as a measure of aggressive behavior. The results show that the prosocial video game group (compared to the neutral video game group) displayed smaller P300 amplitudes, were more accurate in distinguishing violent words, and were less aggressive as evaluated by the CRTT of noise intensity chosen. A mediation analysis shows that the P300 amplitude evoked by violent words partially mediates the relationship between type of video game and subsequent aggressive behavior. The results support theories based on the General Learning Model. We provide converging behavioral and neural evidence that exposure to prosocial media may reduce aggression.

## Introduction

Due to the increasing number of video games and social media sites, people have more opportunities to play digital games than they did in the past. According to a recent investigation, 67% of American families regularly enjoy video games, playing for an average of 8 h per week (Entertainment Software Rating Board, 2012). Additionally, in a large survey of American teenagers (*N* = 1102), 99% of boys and 94% of girls reported playing video games (Lenhart et al., 2008). Moreover, both the total number and average age of video game players is increasing along with the amount of time spent playing (Entertainment Software Association, 2013). Recent meta-analyses have revealed that exposure to violent video games may induce aggressive behavior and inhibit prosocial behavior, while prosocial video games might have the opposite effect (Anderson et al., 2010; Greitemeyer and Mügge, 2014). However, little is known about the neural correlates underlying the effect of prosocial video games on aggression. This study aimed to explore the effect of short-term exposure to prosocial video games on aggressive behavior and to characterize the neural correlates of the relationship between prosocial video games and response to violent words.

### The effect of video games on aggressive behavior

In the early stages of gaming research, the effect of violent video games on aggression received much attention (Griffiths, 1999; Anderson and Bushman, 2001; Sherry, 2001). Exposure to violent video games was found to result in desensitization to violence and increased aggression (Bartholow et al., 2006; Carnagey et al., 2007; Engelhardt et al., 2011). It was postulated that aggressive behaviors were induced through an increase in aggressive attitudes, cognition, and beliefs (Anderson and Bushman, 2002; Greitemeyer and McLatchie, 2011). The general aggression model (GAM; Anderson and Bushman, 2002), which was discussed in the aforementioned studies, states that under the influence of personality variables (e.g., trait aggression) and situational variables (e.g., exposure to media violence), individuals will develop aggressive thoughts and emotions and experience a state of physiological arousal that ultimately leads to aggressive behavior.

Influenced by the recent positive psychology trend, researchers have begun examining the beneficial effects of prosocial video games, especially their potential inhibiting effects on aggression. According to behavioral research, exposure to prosocial video games not only can decrease aggressive cognition (Greitemeyer and Osswald, 2009), but may also rein in aggressive emotions, hostile attributions, and aggressive behaviors (Gentile et al., 2009; Greitemeyer, 2011; Greitemeyer and Osswald, 2011; Greitemeyer et al., 2012). These studies were based on the General Learning Model (GLM; Buckley and Anderson, 2006; Swing et al., 2008), which was derived from GAM. The GLM consists of three approaches (i.e., cognition, emotion, and arousal) to behavior change, through which individuals can modify their behavior under the influence of situational and personality variables (Gentile et al., 2009, 2014). The GLM emphasizes the importance of media content, especially prosocial media, and suggests that exposure can decrease aggressive actions (Gentile et al., 2009, 2014; Greitemeyer, 2011). However, it is still unclear how prosocial video games curb aggression, and this topic has received only limited exploration from a neuroscientific perspective.

### Neural correlates of violent games' effect on aggressive behavior

Although there is a lack of research investigating the effect of prosocial video games on aggressive behavior, several studies have been conducted on the neural correlates of violent video games' effect on aggression. According to research using electroencephalography (EEG) and event-related potentials (ERPs), exposure to violent video games affects the neural mechanisms involved in emotion regulation (Bailey et al., 2011), and chronic exposure to media violence causes desensitization to violence, which can further induce aggression (Bartholow et al., 2006; Engelhardt et al., 2011). Other studies have revealed that short-term exposure to violent video games causes sensitization to violent cues (Staude-Müller et al., 2008; Zhong et al., 2013). In addition, functional magnetic resonance imaging (fMRI) research has shown that during exposure to a violent video game, the prefrontal lobe, frontal-parietal network (left lateral orbitofrontal cortex, right cuneus, and bilateral inferior parietal lobules), and parietal lobe become less active, resulting in a desensitization to violence, a decline in cognitive control, and augmentation of aggressive behavior (Weber et al., 2006; Kelly et al., 2007; Wang et al., 2009; Hummer et al., 2010; Kalnin et al., 2011; Strenziok et al., 2011).

### The P300, video games, and aggressive behavior

The P300 is a clear index of human attention, feeling, and cognition, and arises in the parietal and occipital lobes during information processing. This component can be stimulated using the two-stimulus oddball paradigm, which comprises two types of stimuli: (1) target stimuli that appear occasionally, in this case prosocial or violent words; and (2) non-target stimuli that appear frequently, such as neutral words. When the probability of the target stimuli appearing is low, P300 waves increase dramatically (Mathias and Stanford, 1999; Debener et al., 2005).

Previous research has shown that smaller P300 amplitudes are associated with aggression and chronic violent video game exposure (Bartholow et al., 2006; Engelhardt et al., 2011). Bartholow et al. (2006) used violent, neutral, and negative non-violent images as the stimuli in an ERP task, and revealed that subjects in a chronic violent video game exposure group demonstrated smaller P300 amplitudes than a non-violent video game group. The authors inferred that desensitization to violence is caused by chronic violent video game exposure. Engelhardt et al. (2011) also found that neural desensitization (smaller P300 amplitudes) to violence predicts increased aggression following violent video game exposure, as well as a mediating effect of desensitization to real-life violence (P300) on the increase in aggression that occurs after playing a violent relative to a non-violent video game.

However, some investigations report that short-term exposure to video games can result in greater sensitivity to violence (as opposed to desensitization) and an increase in P300 amplitude compared with exposure to neutral video games (Zhong et al., 2013). In addition, aggressive-cognition triggering has been associated with larger P300 amplitudes in individuals with no history of aggression (Fanning, 2011), and fMRI research has revealed that the dorsal anterior cingulate cortex (dACC) activates in dangerous and aggressive circumstances or in situations involving weapons (Denson et al., 2009). Murray et al. (2006) found that watching violent TV programs resulted in the activation of brain regions involved in emotional adjustment, arousal, attention, and the encoding and retrieval of emotional memory, as well as regions associated with visual movement, viewing objects and scenes, and auditory monitoring. Thus, from these studies, it appears that sensitivity to violence and the specific activation of these brain regions after short-term exposure to violent media were likely related to increases in aggressive cognition.

Prior research has shown that using prosocial and violent words as target stimuli in the oddball paradigm can elicit clear P300 waves (Debener et al., 2005; Nieuwenhuis et al., 2005), and violent words might induce larger P300 waves than prosocial words because of a “negative effect” (Surguy and Bond, 2006; Thomas et al., 2007), which means that negative stimuli produce larger P300 waves. Additionally, according to previous research on the short-term effects of violent video games on aggression (Zhong et al., 2013), brief exposure to a violent video game might activate areas of the brain associated with aggression and produce larger P300 waves.

However, little is known about prosocial video games' effect of this phenomenon, especially in the short term. In the current study, we aimed to build on past research in which the P300 was most affected by video game experience. We sought to characterize the neural correlates underlying the specific effects of prosocial video games in comparison to related research on violence desensitization and aggressive cognition.

According to the GLM, prosocial video games restrain aggression by attenuating the connection between aggressive cognition and aggressive behavior; in other words, prosocial video games reduce aggression by inhibiting aggressive cognition (Greitemeyer and Osswald, 2009), but have no direct relationship with desensitization. Therefore, prosocial video game exposure is likely associated with less deep cognitive neural processing of violent scenarios. Thus, if prosocial video game players encounter a violent context (e.g., violent words or pictures), their neural activation might be decreased; specifically, P300 amplitudes might be smaller compared to people who do not play prosocial video games.

### The present study

This study explored the effect of exposure to prosocial video games on inhibiting aggressive behavior using ERP. Two games were designed for the current study based on previous investigations (Greitemeyer and Osswald, 2010): a prosocial video game and a neutral game without any prosocial components. The influence of these two types of games was then examined using a two-stimulus lexical decision task based on the oddball paradigm. A competitive reaction time task (CRTT) was used to evaluate aggressive behavior in subjects after short-term exposure to the video games. We compared the P300 amplitudes of the two groups during the lexical decision task and analyzed a mediation model. Based on GLM, This model includes game type as the independent variable, aggressive behavior as the dependent variable, and aggressive cognition (i.e., P300 amplitude in response to violent words) as the mediating variable.

We predicted that after subjects played a prosocial video game for a short time, their judgments of violent words would reflect restrained aggressive cognition and diminished P300 amplitudes compared to participants who had played a neutral video game. In terms of behavior, we predicted that playing a prosocial video game would lead to lesser aggressive behavior in the CRTT. According to the GLM, aggressive cognition is a mediating factor in the negative association between prosocial video game playing and aggressive behavior; therefore, we predicted that P300 amplitude (in response to violent words) would mediate the predicted relationship between video games and aggressive behavior.

## Materials and methods

### Participants

Participants included 55 students (27 female and 28 male) from the Southwest University of China. Students were between the ages of 18 and 24, with an average age of 20.76 years (*SD* = 1.76). At the end of the study, subjects were paid RMB 40 and thanked for their time.

In the neutral video game group, there were 28 participants (13 females and 15 males), with an average age of 20.34 years (*SD* = 1.52, range 19–23); however, data from three of the participants were found to contain large movement artifacts during the ERP analysis and therefore were discarded. Thus, in the final analysis of the neutral video game group, data from 25 participants (12 male) were used. In the prosocial video game group, there were 27 participants (14 females and 13 males), with an average age of 20.78 years (*SD* = 1.82, range 18–24). Again, three of the participants' ERP data contained large artifacts and were discarded; thus, the data from 24 participants (12 male) in the prosocial video game group was used for analysis. There was no difference in age between the groups [*t*_(47)_ = 0.15, *p* = 0.88]. The Buss Perry Aggressive Questionnaire (BPAQ; Buss and Perry, 1992) was administered to measure baseline aggressive traits; there was again no difference between the groups [*t*_(47)_ = 0.16, *p* = 0.875]. All subjects were right-handed and had normal or corrected-to-normal vision; no subjects suffered from any neurological defects or damage, such as language impairment, nor had any taken medications recently.

### Ethics statement

This study and the recruitment of subjects were approved by the ethics committee of Southwest University of China. All subjects participated in the study voluntarily and signed informed consent before taking part. The experimental procedure was conducted in accordance with the Helsinki guidelines as per the World Health Organization (World Medical Association, 2000).

### Materials

#### Video games

Two video games were tested in a preliminary study: *Rescue Team 2*, a prosocial game, and *Road Rush*, a neutral game (which was rated according to previous research; Wei et al., 2010). In *Rescue Team 2*, players controlled rescue team members who had to re-construct three islands hit by a hurricane. Tasks included removing wreckage to rescue trapped civilians; repairing a damaged restaurant, house, and lumber mill to produce resources; fixing a bridge, a parking tarmac, and a dock to restore infrastructure; competing against time to collect precious gems; putting out a fire; and rescuing stranded swimmers. *Road Rush* required players to drive a car to the end of the road before their fuel ran out to win the game; it was used as a neutral video game in previous study (Wei et al., 2010).

Several measures were administered to participants after completion of the games. These included the *Game Evaluation Questionnaire* (Anderson and Dill, 2000), in which subjects scored each of the games on six dimensions (frustration, difficulty, enjoyment, proficiency, prosocial content, and aggressive content) using a 5-point rating scale ranging from 1 to 5. Subjects were also required to judge the degree of prosocial and violent actions in each game on a 5-point Likert scale. The *Positive and Negative Affect Schedule* (PANAS, Watson et al., 1988) was used to assess subjects' emotional state after completing the games. The PANAS is divided into positive (PA) and negative (NA) affect subscales, each containing 10 adjectives. Respondents are asked to rate the extent to which they have experienced each particular emotion on a 5-point scale (1 = “not at all” to 5 = “very much”). Finally, the Perceived Arousal Scale (PAS; Anderson et al., 1995) was used to assess subjects' current level of arousal using a list of 31 adjectives.

Thirty-four subjects who were not part of the main experiment were included in the preliminary game evaluation. These subjects were told that the present experiment was intended to aid in the selection of video games for a future study. Subjects were randomly assigned to play one of the two aforementioned video games, and they were asked to answer some questions about their experiences after playing. Before the formal start of the experiment, both groups watched a short film of natural scenery for 1 min in order to establish a uniform baseline level of affect. The subjects played their respective games for 3 min as a practice round to ensure that they understood the rules. Subsequently, subjects played their games for 20 min (official round). When the time was up, the experimenter stopped the subjects and asked them to complete the Game Evaluation Questionnaire, PANAS, and PAS. Table 1 presents the means and standard deviations of affect, arousal, frustration, difficulty, enjoyment, proficiency, prosociality, and violence experienced during both video games.

**Table 1:** ***F*-test for every dimension of different video games**.

**Games**	**Rescue team 2**	**Road rush**	***F*_(1, 33)_**	***p***	**η^2^**
PA	3.69(0.58)	3.42(0.44)	2.43	0.13	0.07
NA	1.41(0.37)	1.49(0.43)	0.37	0.55	0.01
Arousal	4.19(0.32)	4.06(0.35)	1.32	0.26	0.04
Difficulty	3.53(0.72)	3.06(1.13)	2.38	0.12	0.08
Enjoyment	4.35(0.61)	4.17(0.62)	0.81	0.37	0.02
Proficiency	3.18(1.02)	3.17(1.04)	0.01	0.98	0.01
Prosocial content	4.41(0.51)	2.06(0.99)	76.04	<0.01	0.70
Aggressive content	1.29(0.47)	1.44(0.51)	0.82	0.37	0.02
Prosocial action	3.59(0.71)	1.72(0.89)	46.23	<0.01	0.58
Aggressive action	1.18(0.39)	1.39(0.61)	1.49	0.21	0.04

A one-way analysis of variance (ANOVA) was conducted to compare the two games across the various subjective measures. As shown in Table 1, the two video games did not differ in terms of affect (e.g., PA and NA) or arousal (*p* > 0.05). The games also received similar ratings on difficulty, enjoyment, proficiency, and level of violence. However, there was a significant difference between the games from a prosocial perspective, not only in regards to prosocial content but also in the prosocial actions required of the player (*p* < 0.01), with prosocial content and actions in the prosocial video game exceeding those in the neutral game. This suggests that the games used in this study successfully distinguished the prosocial and neutral environments and that these two video games were well matched for affect and arousal. Thus, *Rescue Team 2* was used as the prosocial video game and *Road Rush* as the neutral game in the main experiment.

#### Prosocial, neutral, and violent words

For the second part of the preliminary study, 60 words (two-character word length; 20 each with aggressive, prosocial, and neutral content) were selected from the *Modern Chinese Dictionary* (Wang et al., 1986) for the lexical decision task. Forty subjects who were not included in either the main experiment or the video game evaluation were asked to evaluate the understandability, universality, and aggressive and prosocial characteristics of these words on scales ranging from 1 to 7. The frequency of these words was obtained from the Modern Chinese Corpus (2010)^1^. Table 2 shows that there was no difference in the understandability, universality, or frequency of the words (*p* > 0.05), but that they did significantly differ in terms of aggression and prosocial characteristics (*p* < 0.01). Specifically, prosocial words were rated higher on prosocial characteristics than were the neutral and aggressive words, while the aggressive words were rated higher on aggression than were the neutral and prosocial words (Table 2).

**Table 2:** **A comparison of subjective ratings of the three word types**.

**Variables**	**Prosocial words**	**Neutral words**	**Violent words**	***F*_(2, 80)_**	***p***	**η^2^**
Understandability	6.20(0.81)	6.29(0.88)	6.09(0.74)	1.99	0.14	0.05
Universality	5.59(0.93)	5.61(0.87)	5.36(0.96)	1.59	0.21	0.04
Frequency	14.35(9.89)	12.21(11.28)	8.90(11.73)	1.25	0.29	0.04
Aggressive	1.57(0.74)	1.64(0.64)	6.03(0.55)	899.52	<0.01	0.97
Prosocial	5.93(0.87)	2.93(1.44)	1.72(0.76)	196.23	<0.01	0.83

### Experimental tasks

**Figure 1:**
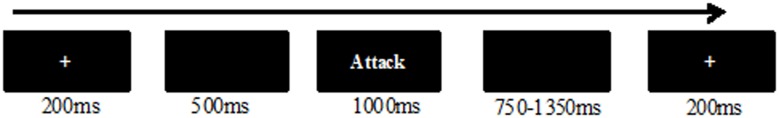
**Lexical decision task trial procedure**.

The lexical decision task, based on the oddball paradigm, consisted of 5 blocks of 200 trials each (20 trials included violent words and 20 trials included prosocial words). The process for each trial is displayed in Figure 1. First, a “+”was displayed for 200 ms, followed by a black screen for 500 ms. Next, the stimulus and reaction screens were presented for 1000 ms. During this time, the subjects had to evaluate each word by pressing a button on the keyboard, with prosocial words represented by the “c” key and aggressive words by the “m” key; no button press was required for neutral words. Stimuli did not vanish until the end of the 1000 ms, even after the button was pressed. After the subject's reaction was recorded, another black screen was displayed for 750–1350 ms. Every third to fifth neutral word trial was followed by either an aggressive or a prosocial word trial. The entire task lasted approximately 50 min, and subjects were given a short rest after completing each block in the task.

The CRTT (Taylor, 1967; Anderson and Bushman, 1997) consisted of two stages. In the first stage, subjects were informed that they would be participating in a reaction time competition against another subject (a virtual subject), in which the slower participant would be punished with a loud burst of sound. Sound intensities ranged from weak to strong in increments of 10 db spanning from 0 to 100 db. Suitable sound intensity for the ear ranges from 80 to 90 db, with an intensity of 100 db or higher leading to slight earache and headache. Thus, the largest sound intensity used was below 100 db in order to avoid pain or a physiological reaction.

During the task, subjects had to respond within 2000 ms; if they did not respond in time, a random sound punishment was given. Additionally, during the first stage of the task, winners were decided randomly, with the loser receiving a random sound burst. This stage was intended to familiarize the subjects with the experimental procedure, as well as to expose them to the different sound intensities. The task was designed to allow participants to hear each of the different sound intensities before its completion (Anderson and Bushman, 1997).

In the second stage, the procedure was the same except subjects were told that they could select any of the 10 sounds to punish their rival if they won the game. When the subject won, the numbers 1 through 10, corresponding to the sound punishment grades, appeared on the screen, and the subjects were permitted to choose the selected punishment for their rival. Conversely, if the subject lost the game, the standby screen emerged and the program assigned a random punishment sound. The noise intensity chosen and the reaction time of the subjects were used as indexes of aggressive behavior.

### Experimental procedure

First, subjects were informed that they were taking part in an EEG/ERP experiment about the effect of video games on attentional bias. After application of the EEG helmet and electrode jelly, subjects were randomly assigned to play either the prosocial or the neutral game for 20 min. After the game, subjects were given a 2-min break before performing the lexical decision task. Subjects were given another 2-min break before they were asked to complete the CRTT. The CRTT required subjects to wear earphones at all times; thus, before the CRTT, the earphones were adjusted to ensure that all participants were using the same model set at the same volume.

### ERP recording and data processing

Continuous EEG recordings were taken using 64 Ag/AgCl unipolar leads on a 64-lead connection system developed by Brain Products GmbH. Electrode placement was based on the International 10–20 system to record the horizontal and vertical electro-oculograms (HEOG and VEOG, respectively) of the right eye. The prefrontal electrode (in the mid-point between FPz and Fz) was connected to the ground, with the prefrontal FCz electrode as the online reference. With an A/D sampling frequency of 500 Hz, the impedance of electrode and scalp was lower than 5 kΩ.

Offline analysis was conducted on the EEG data using Analyzer 2.0 software, with the connection of two earlobes used as re-references. A regression method was applied to remove the HEOG and VEOG from the Analyzer 2.0 system. With a filtering band pass of 0.01–30 Hz and a corrected baseline of −200 ms, this method successfully eliminated artifacts with amplitudes exceeding ±80 μV. Using only correct trials, an epoch of −200–1000 ms and a baseline of 200 ms before stimulus onset was established. The P300 was analyzed according to previous studies (Bartholow et al., 2006; Engelhardt et al., 2011; Zhong et al., 2013); larger P300 waves appear in the parietal lobe, such that if three electrodes—P3, Pz, and P4—were selected for analysis, a large proportion of obvious P300 components would be acquired. Thus, using a P300 amplitude range of 300–800 ms (Bartholow et al., 2006; Engelhardt et al., 2011; Zhong et al., 2013) allowed for the successful extraction of P300 amplitude and behavioral data for analysis.

Behavioral data were analyzed using SPSS version 20.0. A repeated measures ANOVA and tests of simple effects for interactions were conducted. Bootstrapping was applied to test the significance of aggressive cognition (i.e., P300 amplitude in response to violent words) as a mediator, with 1000 resamples and a confidence interval of 95% (Preacher and Hayes, 2008).

## Results

### Behavioral results

#### Lexical reaction results

**Figure 2:**
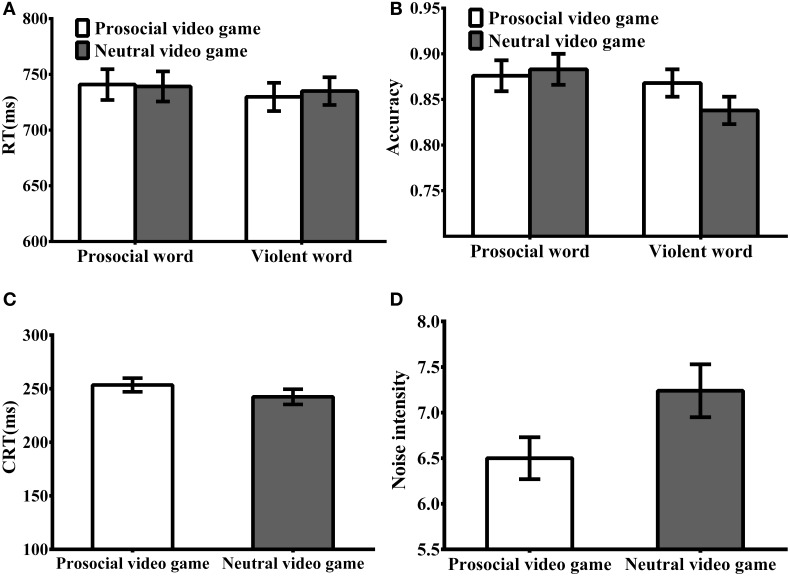
**Behavioral results. (A)** Reaction time for Lexical decision task. There was no main effect of game type and word type or interaction. **(B)** Accuracy for Lexical decision task. Participants were higher accuracy for prosocial words relative to violent words. Once again there was no effect of game type; however, there was a reliable interaction such that people were more accurate at identifying the violent words and this effect was exacerbated after short-term exposure to a prosocial video game. **(C)** Reaction time for CRTT. There was no effect of game type. **(D)** Noise intensity for CRTT. Participants were in the prosocial video game group assigned lower-intensity noise to their rivals compared to the neutral video game group. Error bars were ±1 standard error. Because no reaction for neutral words, Once again they were not included in the behavioral results.

Figure 2 displays the statistics for behavioral results. A 2 × 2 repeated measures ANOVA [game type (prosocial, neutral) × word type (prosocial, aggressive)] was conducted. In terms of reaction time (see Figure 2A), no statistical differences were found for the main effects of game type [*F*_(1, 47)_ = 2.58, *p* = 0.12, η^2^ = 0.05], word type [*F*_(1, 47)_ = 0.53, *p* = 0.47, η^2^ = 0.01]. The interaction of game type × word type was not statistically significant [*F*_(1, 47)_ = 0.63, *p* = 0.43, η^2^ = 0.01].

In terms of accuracy (see Figure 2B), the main effect of game type was not statistically significant [*F*_(1, 47)_ = 1.72, *p* = 0.19, η^2^ = 0.03]; however, the main effect of word type did reach statistical significance [*F*_(1, 47)_ = 7.61, *p* = 0.008, η^2^ = 0.14], with higher accuracy found for prosocial words relative to violent words. Additionally, the interaction of game type × word type was statistically significant [*F*_(1, 47)_ = 4.31, *p* = 0.043, η^2^ = 0.08]. Tests of simple effects revealed that the prosocial video game group showed higher accuracy for violent words than did the neutral group [*F*_(1, 47)_ = 5.19, *p* = 0.02, η^2^ = 0.10], but there was no statistical difference for prosocial words (*F* < 1). Put another way, in the neutral video game group, accuracy for prosocial words was higher than for violent words [*F*_(1, 47)_ = 11.93, *p* < 0.01, η^2^ = 0.21], while there was no statistical difference in the prosocial video game group (*F* < 1).

#### CRTT results

An ANOVA was conducted to examine the behavioral data from the CRTT including competitive reaction times and noise intensity. There was no statistical difference in competitive reaction time between the prosocial and neutral game groups [*F*_(1, 47)_ = 1.31, *p* = 0.26, η^2^ = 0.03] (see Figure 2C). There was a marginally significant difference in aggressive behavior (i.e., noise intensity) between the two game groups [*F*_(1, 47)_ = 4.02, *p* = 0.051, η^2^ = 0.08], such that the prosocial game group assigned lower-intensity sounds to their rivals compared to the neutral game group(see Figure 2D).

#### P300 results

**Figure 3:**
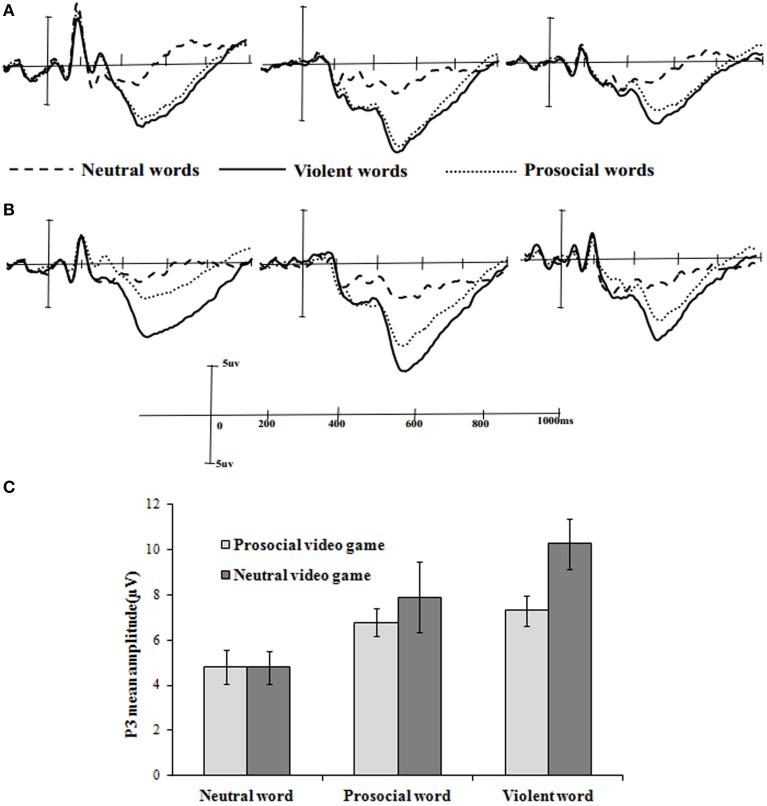
**Prosocial, neutral and violent words reaction of P300. (A)** It illustrates the stimulus-locked grand-averaged ERP waveforms (P300) of prosocial video game played (from left to right: P3, Pz, and P4), with three conditions of reaction by prosocial, neutral and violent words. **(B)** It illustrates the stimulus-locked grand-averaged ERP waveforms (P300) of neutral video game played (from left to right: P3, Pz, and P4), with three conditions of reaction by prosocial, neutral, and violent words. **(C)** P300 results for prosocial and neutral video game playing. There was an interactive effect of game type and word type. P300 amplitude was smaller in the prosocial video game group for violent words compared to the neutral video game group; however, no significant effect was found for neutral and prosocial words. P300 amplitude on the Y-axis was calculated by averaging of Pz, P3, and P4 electrodes readings. Once again error bars were ±1 standard error.

Initial inspection of the waveforms confirmed that the P300 elicited by violent or prosocial words in the oddball task was largest at parietal (P3, Pz, P4) electrode sites (Figures 3A,B). Therefore, the P300 was measured as the average voltage from 300 to 800 ms post-stimulus at the parietal electrodes. A 3 (word type: neutral, prosocial, and violent words) × 2 (game type: prosocial and neutral video games) factorial ANOVA was conducted. The main effect of word type was statistically significant, with neutral (*M* = 4.81, *SD* = 0.52), prosocial (*M* = 8.09, *SD* = 0.67), and violent words (*M* = 8.80, *SD* = 0.66) differing significantly [*F*_(2, 94)_ = 26.17, *p* < 0.01, η^2^ = 0.35]. This finding is consistent with previous research (Surguy and Bond, 2006; Thomas et al., 2007). The main effect of game type was no significant difference [*F*_(1, 47)_ = 3.16, *p* = 0.08, η^2^ = 0.06]. The interaction between word type and game type was also statistically significant, [*F*_(2, 94)_ = 3.82, *p* = 0.03, η^2^ = 0.08]. Simple effects test were conducted. There were no statistical differences in P300 amplitude between neutral (*F* < 1) and prosocial words [*F*_(1, 47)_ = 2.65, *p* = 0.084, η^2^ = 0.07], but P300 amplitude was smaller in the prosocial video game group for violent words [*F*_(1, 47)_ = 5.01, *p* = 0.03, η^2^ = 0.10] compared to the neutral game group(see Figure 3C). Put another way, in the neutral video game group, P300 amplitude for prosocial words was lower than for violent words (*p* < 0.01), while there was no statistical difference in the prosocial video game group (*p* > 0.05).

#### Mediation model analysis

**Figure 4:**
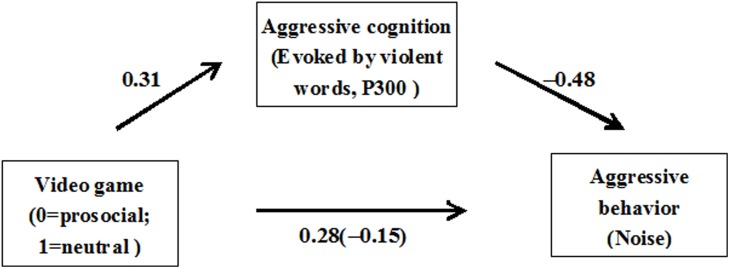
**Mediating effect of aggressive cognition (P300) on video game to predict aggression**.

To further explore the mechanism through which video games inhibited aggressive behavior, the proposed mediation model was analyzed using a bootstrapping procedure (MacKinnon et al., 2007). As shown in Figure 4, the direct effect of video game type on aggressive behavior was statistically significant (*ß* = 0.43,*t* = 3.26, *p* < 0.01), the effect of video game type on violent word recognition (P300) was statistically significant (*ß* = 0.31, *t* = 2.23, *p* = 0.03), and the relationship between violent word recognition (P300) and aggressive behavior was also statistically significant (*ß* = −0.48, *SE* = 0.12, 95% *CI* [−0.74, −0.22]). In addition, the indirect effect of video game type on aggressive behavior (via violent words) was statistically significant (*ß* = −0.15, *SE* = 0.07, 95% *CI* [−0.32, −0.02]). The total effect was 0.28, which contains indirect (−0.15) and direct effects (0.43). P300 amplitude in response to violent words was found to partially mediate the relationship between game type and aggressive behavior (i.e., chosen noise intensity).

## Discussion

This study revealed that short-term exposure to prosocial video game (as compared to neutral video game) lowered aggressive behavior and P300 amplitude in response to violent words but not in response to prosocial or neutral words. Additionally, according to our mediation analysis, aggressive cognition (i.e., P300 amplitude in response to violent words) partially mediates the relationship between video games exposure and aggressive behavior. The results of this study support the GLM hypothesis (Buckley and Anderson, 2006; Gentile et al., 2009, 2014), which argues that short-term exposure to prosocial video games can inhibit aggressive behaviors for a short period by lowering aggressive cognition—namely, P300 amplitudes in response to violent words.

### Inhibition of aggressive behaviors

Our behavioral data revealed a significant difference in the noise intensity selected during the CRTT between prosocial and neutral video games. This result suggests that short-term exposure to prosocial video games can contribute to the inhibition of aggressive behavior, thus confirming findings from previous studies (Greitemeyer and Osswald, 2009, 2010). For example, Greitemeyer and Osswald (2009) found that short-term exposure to a prosocial video game (*Lemmings*) significantly decreased aggressive behaviors, thoughts, and affect compared to a neutral game (*Tetris*). Additionally, in a study testing the GLM, Greitemeyer and Osswald (2010) suggested that short-term exposure to a prosocial video game could increase prosocial behavior via enhancement of prosocial thought. Therefore, it may be inferred that the short-term inhibiting effect of prosocial video games on aggressive behavior was replicated in the present study.

In terms of reaction time, there was no difference in the current study in either reaction time during the CRTT or in the time taken during subjects' evaluation of prosocial and violent words. During the competitive experiment, subjects were informed that they would compete against another subject in terms of reaction time and that they had to react as fast as possible. Initially, we predicted that subjects from the different game groups would have different responses to the competitive process; however, there was no difference, possibly due to the small sample size. In addition, we hypothesized that the evaluation of violent and prosocial words, which represents the processing of social informational cues, would be affected by participation in either the prosocial or the neutral video game. However, there did not appear to be any effect of game type on either of these processes. Thus, it appears that participants' reaction times on both tasks were more reflective of their personal baseline response speeds; short-term exposure to prosocial video games did not affect subjects' reaction times.

However, there was a notable difference in the accuracy of subjects' responses to violent words in the lexical decision task: namely, subjects' accuracy in the prosocial video game group was significantly higher than was that in the neutral video game group. This enhanced identification and judgment of social cues (i.e., violent words) after short-term exposure to a prosocial video game further supports the GLM, which posits that short-term exposure to prosocial games inhibits aggressive behaviors by heightening the accuracy of social cue judgment (Buckley and Anderson, 2006; Gentile et al., 2009, 2014). A similar finding was reported in a previous investigation: highly aggressive individuals made incorrect responses more frequently and were worse at detecting errors than were those with lower levels of aggression (Brazil et al., 2009). Previous research also has found that error detection is closely associated with error-related negativity (ERN) waves, which are induced by error detection and have an onset of 100–200 ms following an error response (van Meel et al., 2007; Olvet and Hajcak, 2008). Because of the limited number of error trials in the ERP task, however, we did not consider the ERN in our analysis, despite it being an ERP component related to aggressive behavior (Brazil et al., 2009; Wiswede et al., 2011). As such, while we cannot claim to have identified a direct link between accuracy and aggressive behavior, the indirect evidence mentioned above suggests that higher accuracy in the judgment of violent cues following prosocial video game exposure helps people to better inhibit cognition associated with these cues and thereby lessen aggressive behavior.

### P300 amplitude decline

This study also revealed that subjects who played the prosocial video game had smaller P300 amplitudes in the presence of violent words (and a weak trend for prosocial words) than did those who played the neutral video game. This is the first time that an effect of prosocial video game exposure on processing of social cues has been documented in a cognitive neuroscience study.

In the current study, prosocial and violent words were novel stimuli; therefore, it is logical that larger P300 amplitudes would be found compared to neutral words. However, subjects in the prosocial video game exposure group had smaller P300 amplitudes in response to violent words (and a weak trend for prosocial words) than did the neutral video game group. This might be because prosocial video game players were less involved in the game than they would be in games with an action context. Prosocial video games usually contain prosocial or helping behaviors—for example, *Lemmings* (Greitemeyer and Osswald, 2010), *Super Mario Sunshine*, and *ChibiRobo* (Gentile et al., 2009) are role-playing games and have less violent action than do other games—but neutral video games have a neutral context, suggesting that they have little effect on social outcomes. Subjects reacted to violent words because they were affected by the prosocial video game context; the game heightened sensitivity to violent words in the word judgment task. Compared to neutral video game players, people in the prosocial video game group required less attention or cognitive resources to differentiate violent words, which might have influenced the amplitude of the P300 (Nieuwenhuis et al., 2005). Hence, short-term exposure to a prosocial video game produced smaller P300 amplitudes in response to violent cues in the lexical decision task.

However, the findings of a previous investigation of the P300 involved in GAM were inconsistent with the findings of the present study; in this previous study, exposure to violent video games decreased P300 amplitude, reflecting desensitization to violence (Bartholow et al., 2006; Engelhardt et al., 2011). In the current study, P300 amplitude was not increased in the prosocial video game group, which would be the predicted opposite effect to violent video games. We believe there are several possible reasons for this finding. First, violent video games increase aggression through desensitization, which is usually a long-term effect. In contrast, there are many findings supporting the notion that short-term exposure leads to increased sensitivity to violence (Kirsh et al., 2006; Staude-Müller et al., 2008; Bailey et al., 2011; Zhong et al., 2013). Although Bartholow et al. (2006) found desensitization to violence and smaller P300 amplitudes after violent video game exposure, they confounded the long- and short-term effects. In other words, the short-term effects of violent video game exposure might be sensitization to violence, while prosocial video game exposure might induce desensitization to violent cues, thereby leading to smaller P300 amplitudes. Second, the current study used Chinese words reflecting cognitive processes; these words might have been more likely to activate aggressive cognition. This differs from the methods employed in previous investigations (Bartholow et al., 2006; Engelhardt et al., 2011), which used pictures indicating emotional processes. Third, exposure to violent video games might activate feelings of disgust, which can induce aggressive behavior and thereby result in a smaller P300 amplitude (see Bartholow et al., 2006; Engelhardt et al., 2011). Those researchers used extremely violent video games (e.g., *Call of Duty*), and did not match affect and arousal between violent and neutral games, which might be related to desensitization. The present study used a prosocial video game that might reduce attentional or cognitive focus on violent cues, and hence smaller P300 amplitudes. As such, short-term prosocial video games exposure does not appear to reduce desensitization, but rather, as per the GLM, might restrain activation of aggressive cognition (i.e., smaller P300 amplitudes).

It is worthwhile noting that P300 amplitudes induced by violent words were not directly associated with accuracy in the behavioral data. This can be due to a number of reasons. First, the accuracy of violent words depended on the corrected and uncorrected responses to violent words, but the P300 represents an average of only correct trial responses to violent words. Second, accuracy has been found to be closely related to the ERN (van Meel et al., 2007; Olvet and Hajcak, 2008), but not the P300. Additionally, an interesting result was that exposure to a prosocial video game lowered the gap in P300 amplitudes between prosocial and violent words, suggesting that the prosocial video game, compared to the neutral video game, “took the edge off” or attenuated reactions to the negative content, resulting in a reduced negative effect of the violent words (Surguy and Bond, 2006; Thomas et al., 2007). However, there is little evidence to support this, meaning that it should be researched in future studies.

### Inhibiting effects of prosocial video games on aggressive behavior

This study also revealed that game type could indirectly influence aggressive behavior via aggressive cognition (i.e., P300 amplitude induced by violent words). Based on this finding, we posit that prosocial video games inhibit individuals' aggressive cognition, resulting in decreased brain activity related to aggressive cognition and P300 amplitude, which in turn leads to the inhibition of individuals' aggressive behavior. Additionally, without constraints on aggressive cognition in long-term memory, individuals playing neutral video games might experience normal activation of their aggression-related cognition under conditions of both normal affect and in the face of environmental or individual cues for aggression. Under these circumstances, both the activity of the nervous system related to aggressive cognition and P300 amplitude will be intensified and individuals' aggressive behaviors will not be restrained. However, this situation did not occur following prosocial video game exposure. Finally, violent words, acting as social cues, invoke aggressive cognition and modify aggressive behavior. We believe that individuals playing prosocial video games experience short-term changes in cognition in the presence of prosocial content, such as performing beneficial tasks in the prosocial video game, which might change the impact of aggressive cues. For instance, playing prosocial video games can decrease attention to violent social cues and thereby curb aggressive behavior. According to GLM, under the influence of external factors (e.g., prosocial media exposure), aggressive behavior can be reduced via inhibition of aggressive cognition or increasing of prosocial cognition (Buckley and Anderson, 2006; Gentile et al., 2009, 2014). It was inhibition of aggressive cognition that acted as a mediator in the relation between video game exposure and aggressive behavior in the current study. However, whether increased prosocial cognition can have the same effect is worth exploring in future research.

### Limitations and future directions

Despite the compelling findings reported herein, this study has several limitations. First, although efforts were made to control for cognition, affect, arousal, enjoyment, difficulty, and other game attributes, various aspects of the prosocial video game used in the study were out of our control. For instance, in previous studies investigating the effects of violent video games on aggressive behavior, the researchers used several different types of violent games, whereas this study focused on only one prosocial video game. Additionally, the prosocial games that are currently available for study, such as *Lemmings*, are often boring and simple. While these prosocial video games have many advantages, violent games are much more prevalent and popular in the video game market. Thus, the content of prosocial video games should be improved in future studies. Second, this study employed a decision task modeled after the oddball paradigm using Chinese characters. To make the subjects focus their attention on the cue words, the words did not disappear after a correct response was made during the task. Although this contributed to the chain reaction of ERP waves, no feedback was presented on the task. Additionally, a habituation effect may have occurred. Third, due to random selection of participants, we did not consider video game experience. Game experience might have been an important factor (Bailey et al., 2010). Future studies should address and control for video game experience. Fourth, our additional results showed a downwards trend for P300 amplitude induced by prosocial words after playing a prosocial video game compared to a neutral video game. We currently are unable to explain this trend, but we believe it relates to lowered attentional resources in response to prosocial video game exposure; still, there is no evidence to support this interpretation. It must be studied in more detail in the future.

Additionally, EEG recordings typically require zero references (Yao, 2001; Tian and Yao, 2013), but existing references, such as the ear connection and the common average references, were used in the current study. Thus, it is important to study the influence of these types of non-zero references on EEG results. Finally, several previous studies have reported that P300 amplitude decreases in response to violent video games, leading to desensitization to violence and intensifying aggression. However, this study showed that short-term exposure to a prosocial video game could inhibit individuals' aggression and lead to a decline in P300 amplitude. This raises questions about the relationship between these effects. For instance, will exposure to prosocial video games result in desensitization to prosocial preferences? Future studies should be undertaken to explore this relationship.

## Conclusions

Despite the limitations, the present study was the first to explore the GLM-based hypothesis of whether there is an inhibitory effect of exposure to prosocial media on individuals' aggression from a neuroscientific perspective. Specifically, the results not only verified the short-term effects proposed by the GLM, but also showed that short-term exposure to a prosocial video game results in a decrease in P300 amplitude in response to violent words. Short-term exposure to prosocial video game can not only directly inhibit aggression and reduce aggressive behaviors, but also indirectly inhibit these through a decrease in aggressive cognition (i.e., P300 amplitude in response to violent cues).

### Conflict of interest statement

The authors declare that the research was conducted in the absence of any commercial or financial relationships that could be construed as a potential conflict of interest.

## Footnotes

^1^Modern Chinese Corpus. (2010). Retrieved May 12th 2014 from http://www.dwhyyjzx.com/cgi-bin/yuliao/search/?k=%C9%B1%CA%D6&c=all.
